# Bifurcated binding of the OmpF receptor underpins import of the bacteriocin colicin N into *Escherichia coli*

**DOI:** 10.1074/jbc.RA120.013508

**Published:** 2020-05-12

**Authors:** Katarina Bartoš Jansen, Patrick George Inns, Nicholas George Housden, Jonathan T. S. Hopper, Renata Kaminska, Sejeong Lee, Carol V. Robinson, Hagan Bayley, Colin Kleanthous

**Affiliations:** 1 Department of Biochemistry, University of Oxford, Oxford, United Kingdom; 2 Chemistry Research laboratory, University of Oxford, Oxford, United Kingdom

**Keywords:** Gram-negative bacteria, outer membrane, porin, bacteriocin, translocation, microscopy, calorimetry, colicin N (ColN,), OmpF, osmoregulation, gram-negative bacteria, fluorescence recovery after photobleaching (FRAP), mass spectrometry (MS), isothermal titration calorimetry (ITC), colicin N

## Abstract

Colicins are *Escherichia coli–*specific bacteriocins that translocate across the outer bacterial membrane by a poorly understood mechanism. Group A colicins typically parasitize the proton-motive force–linked Tol system in the inner membrane via porins after first binding an outer membrane protein receptor. Recent studies have suggested that the pore-forming group A colicin N (ColN) instead uses lipopolysaccharide as a receptor. Contrary to this prevailing view, using diffusion-precipitation assays, native state MS, isothermal titration calorimetry, single-channel conductance measurements in planar lipid bilayers, and *in vivo* fluorescence imaging, we demonstrate here that ColN uses OmpF both as its receptor and translocator. This dual function is achieved by ColN having multiple distinct OmpF-binding sites, one located within its central globular domain and another within its disordered N terminus. We observed that the ColN globular domain associates with the extracellular surface of OmpF and that lipopolysaccharide (LPS) enhances this binding. Approximately 90 amino acids of ColN then translocate through the porin, enabling the ColN N terminus to localize within the lumen of an OmpF subunit from the periplasmic side of the membrane, a binding mode reminiscent of that observed for the nuclease colicin E9. We conclude that bifurcated engagement of porins is intrinsic to the import mechanism of group A colicins.

## Introduction

Colicins are a diverse group of protein toxins deployed by commensal and pathogenic bacteria alike that kill neighboring cells for the producing population to gain a competitive advantage (1, 2). A major challenge faced by all protein bacteriocins is how to cross the outer membrane that is otherwise impermeable to conventional antibiotics such as vancomycin (3, 4). Colicins achieve this feat by contacting one of two energized systems in the inner membrane of *Escherichia coli*; group A colicins exploit the Tol system (also known as Tol-Pal), whereas group B colicins exploit the Ton system (5–7). Both systems are coupled to the proton-motive force, which is thought to drive entry of the colicin across the outer membrane (3, 6, 8–11). To contact these systems, however, colicins have to deposit unfolded epitopes to the periplasm, which typically mimic interactions of endogenous periplasmic protein partners. Studies are beginning to elucidate how such “directed epitope delivery” is achieved (12). The group A colicin ColE9 delivers its unfolded Tol-binding epitope through the pores of OmpF following binding of the colicin to its receptor, the vitamin B_12_ transporter BtuB (13). The group B nuclease pyocin S2, which kills *Pseudomonas aeruginosa* cells, delivers its Ton-binding epitope through the pyoverdine transporter FpvAI by a process that essentially mimics energized entry of the siderophore through the transporter (14). These defined periplasmic entry routes for bacteriocin epitopes remain controversial (15). Here, we investigate these early stages of bacteriocin import for the group A colicin, ColN. We show how ColN binds OmpF, laying the foundations for understanding how all group A bacteriocins penetrate the outer membranes of their target bacterial species.

ColN (∼42 kDa) is one of the smallest colicins known (8, 16). Unlike well-characterized colicins such as E9 and Ia, which have long helical regions that allow binding to their primary receptor (BtuB) at a location distant from their OmpF translocator, ColN is more compact, suggesting that the primary receptor must be located in close proximity to its translocator protein OmpF (17). This requirement for proximate receptor and translocator proteins was fulfilled by early suggestions in the literature that OmpF served as both primary receptor and translocator for ColN (18–21). Subsequent genetic and biophysical studies, however, pointed to lipopolysaccharide (LPS) having an important role in ColN uptake in *Escherichia coli* (22, 23). These studies led to the suggestion that LPS itself is the primary receptor for ColN (8, 17, 24). Here, we re-examine the interactions of ColN with cell envelope components and demonstrate that contrary to the current prevailing view, ColN uses the porin OmpF as both receptor and translocator and that LPS plays a relatively minor role in the import of the colicin.

## Results and discussion

### ColN has distinct OmpF-binding sites

**Figure 1.**
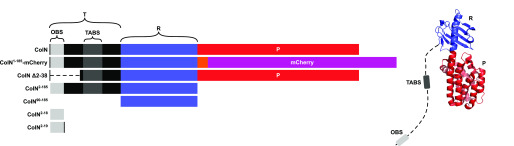
**The structure of colicin N and the constructs used in the present study.** The pore-forming domain is represented in *red* and consists of residues 186–387, the receptor-binding domain is represented in *blue* and consists of residues 90–185, the translocation domain is represented in *black* and consists of residues 1–89, this domain is intrinsically unstructured, hence is not observed in the crystal structure. The TolA-binding box is represented in *dark gray* and consists of residues 44-66 (25), the OmpF-binding site is represented in *light gray* and consists of residues 2-18.

**Figure 2.**
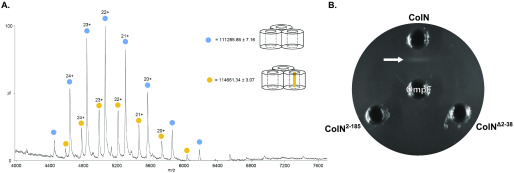
*A,* native MS of OmpF-trypsin digested ColN. *Blue circles*, OmpF; *yellow circles,* OmpF + 3375 Da ColN peptide. *B,* immunodiffusion assay of OmpF and colicin N constructs, four 3-mm wells were cut out of a potassium phosphate buffer, 0.5% agarose gel, pH 6.65, 1% β-OG, 20 µl of each protein was added to the wells at a concentration of 17 μm, plates were incubated overnight at room temperature to allow for precipitation.

**Figure 3.**
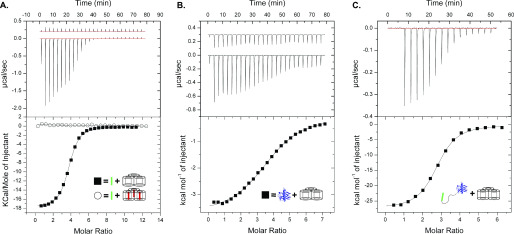
*A,* ITC traces for ColN-OBS (residues: 2-18) OmpF binding (*filled square*) a *K_d_* of 1.4 μm, Δ*H* of −17.9 kcal/mol, Δ*S* of −33.4 cal/mol/K, and *n* of 3.67 peptide/OmpF were determined from this trace, 16.5 μm OmpF was present in the cell, the syringe injected 882 μm ColN^2-19^. ColN-OBS shows no binding to OmpF in samples pre-treated with 1 mm ColE9 OBS1 (residues: 2-18) (*open circle*). 15 μm OmpF and 1 mm ColE9^2-18^ was present in the cell, the syringe injected 882 μm ColN^2-19^. *B,* ITC trace for ColN-R (residues: 90-185) OmpF binding (*filled square*). A *K_d_* of 14 μm, Δ*H* of −3.8 kcal/mol, Δ*S* of −9.6 cal/mol/K, and *n* of 3.82 ColN^90-185^/OmpF were determined from this trace, 39 μm OmpF was present in the cell, the syringe injected 1.3 mm ColN^90-185^. *C,* ITC trace for ColN^2-185^ OmpF binding (*filled square*), in the absence of LPS. A *K_d_* of 214 nm, Δ*H* of −27.7 kcal/mol, Δ*S* of −62.2 cal/mol/K, and *n* of 2.69 ColN^2-185^/OmpF were determined from this trace, 6 μm OmpF was present in the cell, the syringe injected 60 μm ColN^2-185^. All ITC experiments were conducted in 20 mm potassium phosphate, pH 6.5, 1% β-OG.

Structural studies are consistent with ColN being composed of three domains (21) (Fig. 1): an N-terminal, intrinsically disordered domain (residues 1–89; ColN^1-89^) that contacts the periplasmic protein TolA and is involved in translocation (25, 26), a central domain (residues 90–185; ColN^90-185^) implicated in binding an outer membrane receptor (21), and a C-terminal cytotoxic pore-forming domain (residues 186-387; ColN^186-387^) that depolarizes the inner membrane following import to the periplasm (21, 27). Early studies of ColN cytotoxic activity demonstrated a critical requirement for OmpF. *E. coli ompF* deletion strains are resistant to ColN (20), which is a specific interaction given that other closely related outer membrane porins, such as OmpC, are unable to complement this phenotype (19). Indeed, no other OMPs have been identified in its uptake mechanism (22). Subsequent *E. coli* genome-wide screens revealed the importance of LPS on the cytotoxicity of ColN. Knockouts of genes involved in various steps of LPS biosynthesis resulted in ColN resistance, including genes causing deep-rough phenotypes (22). The importance of LPS was further supported by biophysical measurements that suggested that ColN^91-184^ binds LPS with micromolar affinity (23). The N-terminal residues 8-15 of ColN were implicated in binding OmpF (28). The picture emerging from these investigations is one in which ColN engages LPS through its central receptor-binding domain, then recruits a neighboring OmpF using its N terminus that acts as a guide for deposition of a TolA-binding (TAB) epitope into the periplasm for activated import via the Tol system. Colicin OmpF-binding site (OBS) sequences are known to bind within the lumen of OmpF subunits rendering them resistant to proteolysis (12). ColE9 has two OmpF-binding sites within its disordered translocation domain, OBS1 and OBS2. Both sites bind to OmpF within the lumen of individual β-barrel subunits and some OBSs do so from a specific side of the membrane, for example, ColE9 OBS1 binds preferentially from the periplasmic side of the outer membrane (13, 29). We set out to identify the complete OBS of ColN by incubating purified preparations of ColN and OmpF, treating the complex with trypsin, and then identifying ColN fragments that remained bound to the porin by native state MS (MS) (Fig. 2*A*) See “Experimental procedures” for details. We identified a ColN peptide of mass 3,375 Da that remained bound to OmpF trimers in the gas phase. This mass corresponds to residues 2-38 within the disordered translocation domain of the colicin (the N-terminal methionine is usually absent in purified ColN). It should be noted that prior to native MS, upon mixing, ColN and OmpF formed a precipitate that was solubilized upon trypsin treatment. We investigated this precipitation through an immunodiffusion assay (Fig. 2*B*). This assay is analogous to the classical double diffusion Ouchterlony assay used to demonstrate antibody cross-reactivity with antigens in agarose gels. The bivalent nature of antibody interactions with antigens, whereby each individual Fab of the antibody engages a separate antigen, results in aggregation evident in agarose gels as a zone of precipitation. In this assay ColN and OmpF displayed binding induced precipitation. This precipitation was abolished upon removal of either the putative OBS identified by native MS or the pore-forming domain of ColN. It could be possible that this binding induced precipitation occurred due to the presence of multiple OmpF-binding sites on ColN. To investigate this we carried out ITC experiments with different regions of ColN. To further investigate the OBS of ColN we divided the putative sequence into two peptides: ColN^2-19^ and ColN^20-38^, of which only ColN^2-19^ showed binding by MS. ColN^2-19^ bound OmpF in ITC experiments with an affinity of 1.4 μm (Fig. 3*A*), it has been previously reported that ColN^1-92^, representing the intrinsically disordered translocation domain, binds to OmpF with an affinity of 3.4 μm, and this binding is abolished by a F14G mutation (28). A similar affinity has been reported for the OBS1 sequence of ColE9 (12) despite there being little sequence similarity between the two peptides. Moreover, in the presence of 1 mm ColE9 OBS1, ColN^2-19^ binding was completely abolished in ITC experiments (Fig. 3*A*), consistent with this region of ColN binding within the lumen of an OmpF subunit. We conclude that residues 2-19 of ColN house an OBS that binds within the lumen of a single subunit of OmpF. It has been suggested in the literature that the central globular receptor-binding domain of ColN can bind to OmpF (18, 30). To investigate the relative OmpF binding contributions of the OBS and the receptor-binding region of ColN we conducted ITC (Fig. 3), which was possible once the ColN pore forming domain was removed, preventing precipitation. As stated previously, the ColN OBS bound OmpF with an affinity of 1.4 μm (Fig. 3*A*), the receptor-binding domain of ColN (ColN^90-185^) (Fig. 3*B*) bound OmpF with an affinity of 14 μm and ColN with both the OBS and the receptor-binding domain present (ColN^2-185^) bound OmpF with a higher affinity of 214 nm (Fig. 3*C*).

### ColN^2-19^ binds OmpF from the periplasmic side of the membrane

**Figure 4.**
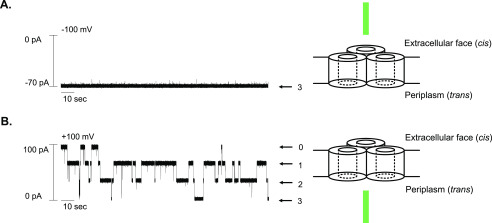
*A,* OmpF single channel conductance in planar lipid bilayers upon addition of 1 μm ColN^2-19^ from the extracellular (*cis*) face of the OmpF channel. (*B*) OmpF single channel conductance in planar lipid bilayers upon addition of 1 μm ColN^2-19^ from the periplasmic (*trans*) face of the OmpF channel. Conductance measurements were performed in 100 mm potassium chloride, 20 mm potassium phosphate, pH 6.5, at room temperature. *Arrows* indicate the different levels of membrane conductance and associated numbers represent the number of open channels associated with conductance level.

OBS1 of ColE9 is known to bind the lumen of an OmpF subunit from the periplasmic side of the membrane. This orientational bias was established by first demonstrating the orientation of OmpF channels in supported phospholipid bilayers (PLBs) (31) and then determining from which side of the membrane OBS1 blocked OmpF ion conductance channels (29). We used a similar approach to determine whether the OBS of ColN had a preferred orientation of binding. No inhibition of OmpF ion conductance was observed when 1 mm ColN^2-19^ peptide was added to the *cis* (extracellular) side of a PLB in which all three OmpF channels were open (Fig. 4*A*). Conversely, when 1 mm ColN^2-19^ was added to the *trans* (periplasmic) side of the PLB, four distinct membrane conductance states were observed (Fig. 4*B*), corresponding to an OmpF trimer in which either none, one, two, or three OmpF channels were blocked by the OBS peptide. Hence, the N-terminal OBS of ColN binds OmpF from the periplasmic side of the membrane similar to that of OBS1 in ColE9.

### LPS increases the affinity of ColN for OmpF

**Figure 5.**
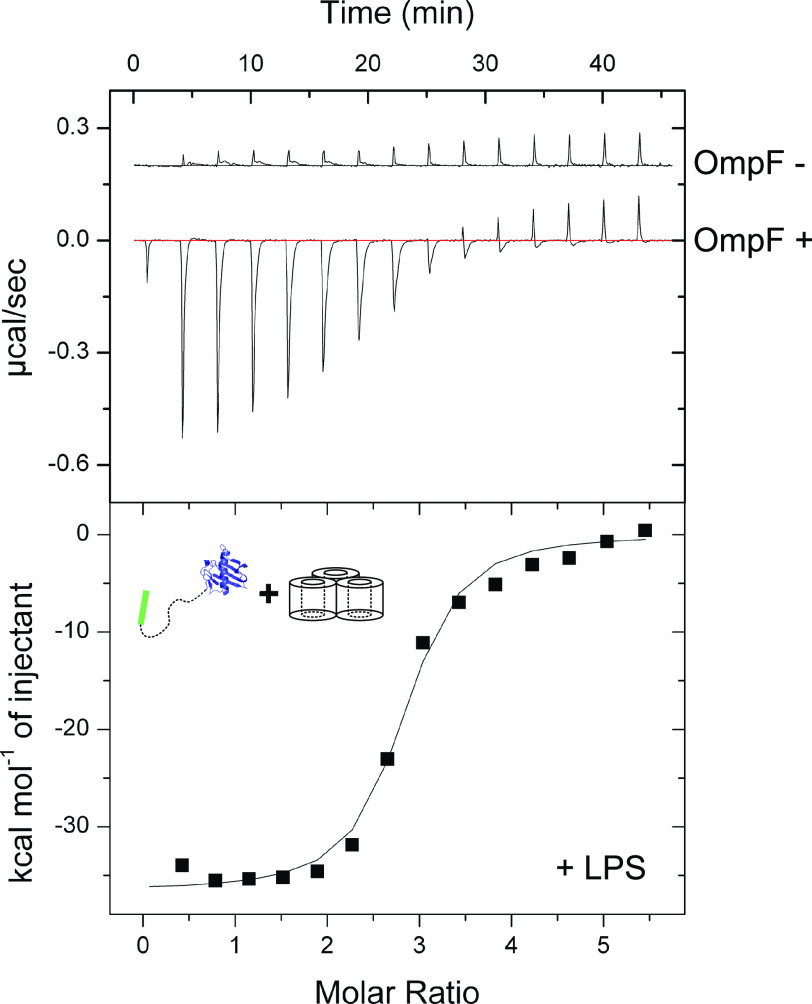
**ITC trace for ColN^2-185^ binding to detergent solubilized B^E^3000 outer membranes (OmpF+) (*filled square*).** From two repeats, an average *K_d_* of 119 nm, Δ*H* of −38.8 kcal/mol, Δ*S* of −98.1 cal/mol/K were determined. The “OmpF−” trace represents ColN^2-185^ binding to detergent-solubilized BZB1107 outer membranes. All ITC experiments were conducted in 20 mm potassium phosphate, pH 6.5, 1% β-OG. Either solubilized B^E^3000 or BZB1107 was present in the cell, the syringe injected 100 μm ColN^2-185^.

Previous studies have shown that ColN^90-185^ interacts with LPS *in vitro*, specifically to three heptose and a single glucose sugar moiety (23), from which it has been concluded that LPS acts as the primary ColN receptor in the OM (8, 17, 24). However, associations with LPS may simply be a reflection of the close association of LPS with OmpF; LPS binds between the subunits of OmpF to stabilize the trimer and is required for folding and insertion of the OmpF trimer into the outer membrane (32, 33). To assess the relative impacts of OmpF and LPS on ColN binding, ITC titrations were performed into detergent-solubilized B^E^3000 outer membranes (OmpF+, OmpC−) and detergent-solubilized BZB1107 outer membranes (OmpF−, OmpC−, LamB−). For these experiments, ColN lacking the pore-forming domain was used (ColN^2-185^) (Fig. 5). Crude OmpF preparations from detergent-solubilized B^E^3000 membranes with abundant LPS bound ColN^2-185^ with an affinity of 119 nm, whereas equivalent BZB1107 membrane extracts containing equivalent amounts of LPS, but no OmpF, showed negligible binding. When compared with ColN^2-185^ binding to purified OmpF lacking LPS (Fig. 3*C*) (verified by SDS-PAGE and LPS staining, Fig. S1*B*), a modestly decreased affinity of 214 nm is observed. It should be noted that the fit observed in Fig. 5 is worse than that of Fig. 3*C,* which could suggest a less significant difference between the *K_d_* values. These experiments demonstrate that OmpF is the primary receptor for ColN but that this binding is modestly improved by LPS bound to OmpF.

### ColN binds exclusively to OmpF in vivo

**Figure 6.**
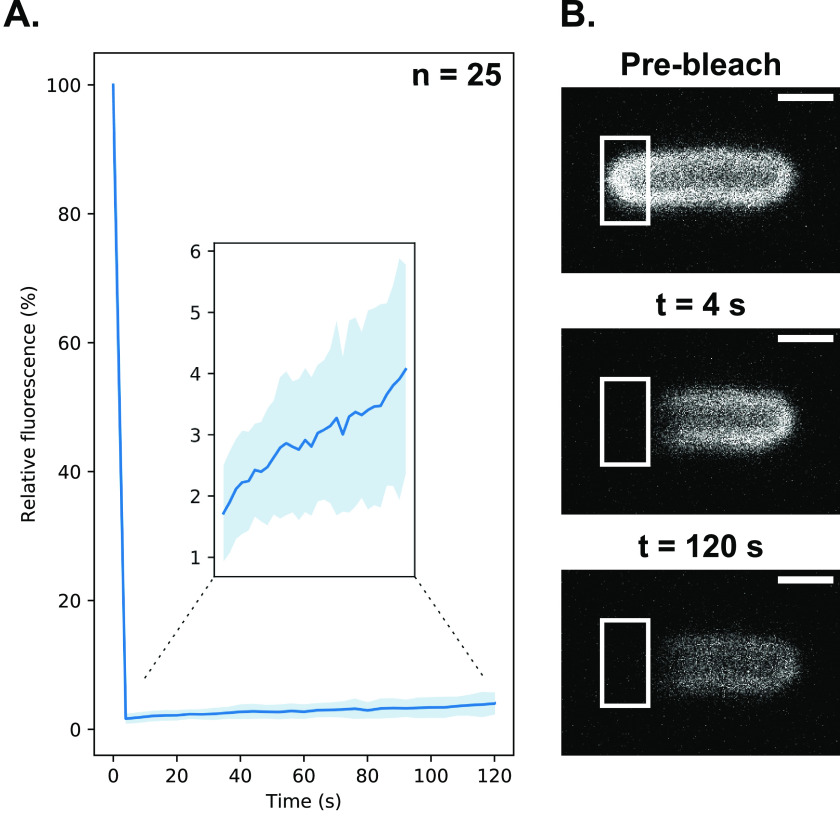
*A,* fluorescence recovery after photobleaching of OmpF after labeling *E. coli* BL21(DE3) with ColN^1-185^-mCherry. The FRAP curve shows the fluorescence recovery over 120 s with imaging conducted every 4 s. After 120 s recovery reached a relative fluorescence value of 4.1 ± 1.7%. *Inset,* FRAP curve from 4 to 120 s. *B,* representative images from one FRAP experiment, the bleaching target area is highlighted, all images were contrast adjusted to the same arbitrary level. All *scale bars* represent a distance of 1 μm.

**Figure 7.**
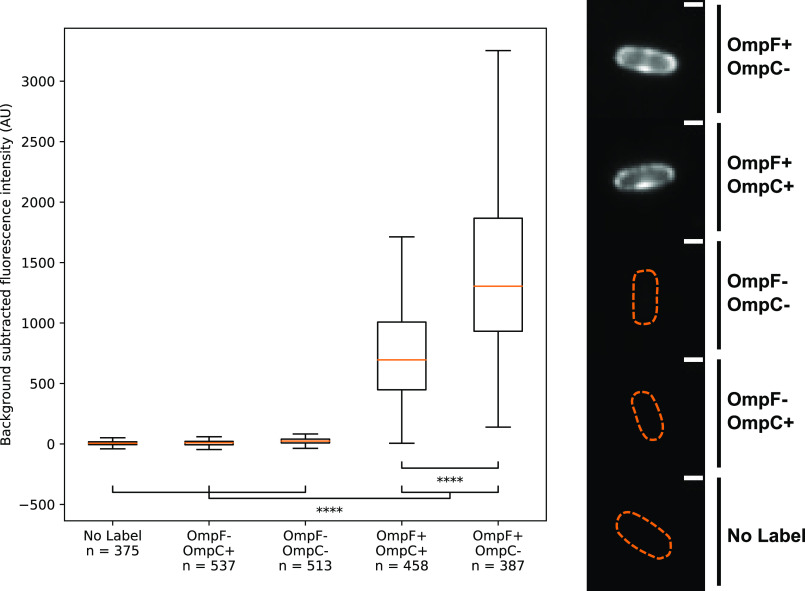
**ColN^1-185^-mCherry labeling fluorescence quantification.** Box and whisker plots showing median fluorescence intensity, upper and lower quartiles, *whiskers* represent the highest and lowest data points within the upper quartile + 1.5 × the inter quartile range, and the lower quartile −1.5 × the inter quartile range. Strains tested were as follows. *No Label,* BW25113 untreated; *OmpF*−, *OmpC*+, JW0912-1; *OmpF*−, *OmpC*−, BZB1107; *OmpF*+, *OmpC*+, BW25113; and *OmpF*+, *OmpC*−, JW2203-1. Single representative images of cells from each condition tested are displayed (*right*), the bounds of these cells were determined from transillumination images and these bounds are represented by the *orange dashed lines*. Cell images were contrast adjusted to the same arbitrary level, all *scale bars* represent a distance of 1 μm.

To establish if the OmpF specificity we had observed *in vitro* reflected the situation *in vivo* we visualized *E. coli* cells by fluorescence microscopy using fluorescently labeled ColN. We have previously developed two types of fluorescence microscopy approaches for labeling bacteriocin receptors in the outer membranes of bacteria, wherein bacteriocins are labeled at their C terminus either with organic dyes, such as Alexa Fluor 488, or fused to fluorescent proteins such as GFP. The former are more photostable but can still be imported into bacteria (14). Fluorescent proteins block bacteriocin import into bacteria but retain the ability of the bacteriocin to bind to outer membrane receptors. We therefore fused mCherry to the C terminus of ColN^1-185^ (substituting the pore-forming domain for mCherry), to generate a fluorescently labeled ColN unable to translocate into *E. coli* (ColN^1-185^-mCherry) (Fig. S2). The abolition of transport was confirmed by fluorescence recovery after photobleaching (FRAP) experiments (Fig. 6). The lack of FRAP is indicative of the bacteriocin remaining bound to its immobile OMP in the outer membrane, as has been observed for other colicins bound to the OMPs BtuB and Cir in *E. coli* (14, 34). If the bacteriocin had translocated to the periplasm, as in the case of the *P. aeruginosa*-specific bacteriocin pyocin S2 labeled with AF488, then fluorescence recovery in FRAP experiments would be observed (14). We next determined the average fluorescence intensity for a large number of cells following the addition of ColN^1-185^-mCherry (200 nm) to different *E. coli* strains (Fig. 7). In cells lacking both OmpF and OmpC (*E. coli* BZB1107) or cells expressing only OmpC (*E. coli* JW0912-1) the level of fluorescence was essentially equivalent to that of the no ColN^1-185^-mCherry control. By contrast, the average fluorescence intensity for cells expressing only OmpF (*E. coli* JW2203-1) was 241-fold above the negative control. Expression of both OmpF and OmpC reduced ColN^1-185^-mCherry binding by approximately half relative to OmpF-only cells. We conclude that ColN binds exclusively to OmpF in the outer membrane of *E. coli*. Moreover, these fluorescence microscopy experiments show unequivocally that LPS is not the receptor for ColN because the bacteriocin does not bind to the extracellular leaflet of *E. coli* where LPS is located unless OmpF is also present.

## Concluding remarks

**Figure 8.**
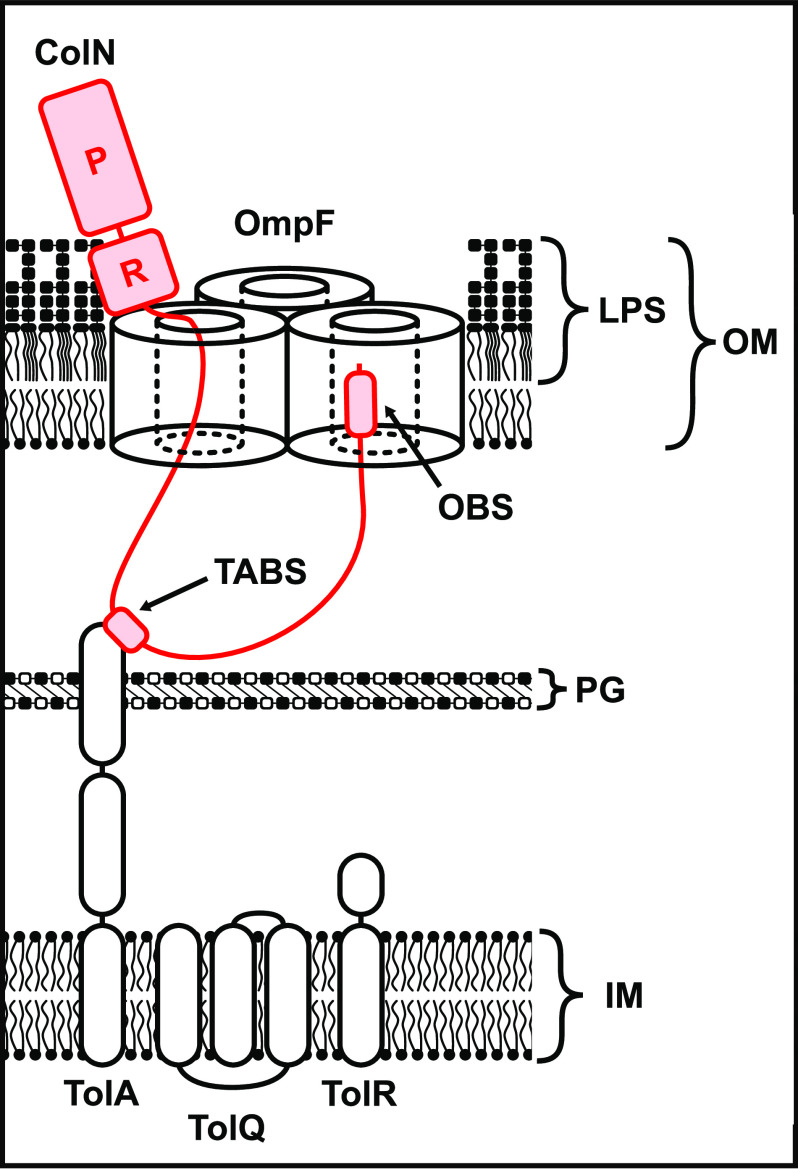
**Our proposed model of colicin N translocation.** The receptor-binding domain of colicin N binds to OmpF (the affinity of which is modestly improved by LPS), the intrinsically disordered translocation domain threads through the lumen of a single OmpF barrel the TolA-binding site (*TABS*) binds to TolA to couple to PMF to energize import. The N-terminal OmpF-binding site (OBS) binds to the lumen of another OmpF barrel within the OmpF trimer.

Through the use of native MS, diffusion-precipitation assays, ITC, PLB single channel conductance measurements, and *in vivo* fluorescence microscopy we have demonstrated that ColN uses OmpF as its OMP receptor and then most likely uses the same trimer molecule as its translocator to the periplasm (Fig. 8). We also show that whereas LPS is not the receptor for ColN, the glycolipid nevertheless modestly improves binding affinity for the porin. Our study also points to a generic mode of porin exploitation by group A colicins (E2-E9, N, A) whereby an N-terminal OBS translocates through one OmpF subunit to bind a neighboring subunit from the periplasmic side of the membrane. In the case of ColE9, the colicin becomes localized to the cell surface through high affinity binding of the vitamin B_12_ transporter BtuB before passing its disordered OBS sequence through OmpF (13). In the case of ColN, the colicin binds directly to the porin via its central receptor-binding domain and then translocates its disordered OBS into the periplasm. In both cases, this form of directed epitope delivery results in binding epitopes for TolB and TolA, respectively, being presented in the periplasm the binding of which activates import of the bacteriocin across the outer membrane (13).

## Experimental procedures

### Plasmids

WT ColN gene was ordered from Eurofins Scientific with incorporated 5' NdeI and 3' XhoI restriction sites. The gene was ligated into pET21a vector (Novagen) adding a noncleavable His_6_ tag at the C terminus of WT ColN. This vector (pNGH237) was used as a parent plasmid to make ColN truncation by mutagenesis PCR with phusion high-fidelity DNA polymerase (New England Biolabs). ColN^1-185^ without its pore-forming domain (missing 186-387 amino acid residues) was designed using the QuikChange (Agilent Technologies) mutagenesis PCR protocol introducing the XhoI site using the following primers: R3 (CTTTTTCATTTCTTTCTTTCTCGAGCTTCCTAAATAATAAAAACTG) and F3 (CAGTTTTTATTATTTAGGAAGCTCGAGAAAGAAAGAAATGAAAAAG). The resulting plasmid was digested with XhoI restriction enzyme and after the linearized plasmid was gel purified, pKBJ11 was generated by ligation using T4 Ligase (New England Biolabs). The gene for the central domain of ColN^91-185^ was generated by PCR on pKBJ11 using the following primers: R3 and F24 (GAGGTACATATGAGTGCTAAGGCTGGAGAG). The PCR product was gel purified, NdeI/XhoI digested, and ligated in to NdeI/XhoI pET21a vector (Novagen) generating pKBJ45. The gene for ColN^1-185^-mCherry was ordered from GenScript with a 13-amino acid linker containing a tobacco etch virus protease site and 5' NdeI and 3' XhoI sites that allowed for digestion and ligation into a NdeI*/*XhoI pET21a vector, generating pKBJ51.

### Protein expression and purification

WT ColN plasmid was transformed into New England Biolabs T7 express lysY/Iq competent *E. coli*. Other ColN constructs were transformed using New England Biolabs *E. coli* BL21(DE3). Truncated ColN constructs were expressed and purified as described in Ref. 35 with the following exceptions: carbenicillin was used in place of ampicillin, induction was conducted with 0.5 mm isopropyl 1-thio-β-d-galactopyranoside immediately after cold shock, expression was conducted overnight at 16 °C. Cells were lysed by sonication and cell debris was pelleted by centrifugation at 20,000 × *g* for 30 min After nickel affinity chromatography the constructs were further purified by size exclusion chromatography. The purification of ColN^1-185-^mCherry was conducted in a similar fashion, with the following exceptions: ampicillin was used in place of carbenicillin and induction was conducted with 1 mm isopropyl 1-thio-β-d-galactopyranoside at 37 °C for 3-4 h. The purification of OmpF was performed according to a protocol described (12) with few exceptions. *E. coli* B^E^3000 was grown overnight at 37 °C in 10 liters of M9 medium (6.8 g/liter of Na_2_HPO_4_, 3 g/liter of KH_2_PO_4_, 0.5 g/liter of NaCl, and 100 mg/ml of NH_4_Cl supplemented with 20% (w/v) glucose, 10% (w/v) HyCase-amino, 2 mm MgSO_4_, 3 μg/ml of Fe(II)Cl_2_, 0.1 mm CaCl_2_. Cells were harvested and re-suspended in 200 ml of 10 mm Tris-HCl and 0.25% (w/v) lithium diiodosalicylic acid, pH 8.0. Upon the addition of 1 mm phenylmethylsulfonyl fluoride, the cells were sonicated and the insoluble cell debris was spun down at 7,000 × *g* for 30 min at 4 °C. Ultracentrifugation steps were carried out as previously (albeit at 4 °C). The OM was extracted in 10 mm Tris-HCl, pH 8.0, 5 mm EDTA, 2% (w/v) octyl β-glucoside (β-OG). This sample was used to obtain ITC measurements for OmpF with LPS present (*E. coli* BZB1107 cells were treated in an identical fashion to obtain the crude OM extract lacking OmpF). To further refine OmpF purification and remove LPS from the sample, the homogenized OM pellet was passed through a Q-Sepharose column (GE Healthcare), extensively washed with 20 mm Tris-HCl, pH 8.0, 5 mm EDTA, 1% (w/v) β-OG, and eluted with a 0-0.5 m gradient of 20 mm Tris-HCl, pH 8.0, 5 mm EDTA, 1% (w/v) β-OG, 1 m LiCl. The sample was buffer exchanged back into 20 mm Tris-HCl, pH 8.0, 5 mm EDTA, 1% (w/v) β-OG using a HiPrep 16/60 Sephacryl 300 high resolution column (GE Healthcare). OmpF without LPS was purified using 1.7-ml Mono Q 4.6/100 PE column (GE Healthcare) using 20 mm Tris-HCl, pH 8.0, 5 mm EDTA, 1% (w/v) β-OG, and a 0–100% gradient of 20 mm Tris-HCl, pH 8.0, 5 mm EDTA, 1% (w/v) β-OG, 1 m LiCl.

### Isothermal titration calorimetry

All ITC experiments were performed using MicroCal ITC_200_ at 25 °C in 20 mm potassium phosphate buffer, pH 6.5, supplemented with 1% (w/v) β-OG. Before the measurements, purified protein samples were buffer exchanged using 5-ml HiTrap desalting columns (GE Healthcare). The sample cell was filled with OmpF trimer at 2-55 μm. ColN constructs were titrated depending on their binding affinity to OmpF in the following concentrations: 60 μm ColN^1-185^ (ColN without the pore forming domain) and 1.3 mm ColN^91-185^ (ColN central domain). The peptides used for the ITC measurements with the following sequences were purchased from Activotec: ColN^2-19^ [(NH_2_)-GSNGADNAHNNAFGGGKN-(CONH2)] and ColE9 OBS1-SB935-2 [(NH_2_)-SGGDGRGHNTGAHSTSG-(CONH2)]. The second batch of ColE9^2-18^ peptide used to generate Fig. 3 was ordered from Severn Biotech Ltd. The peptides were used at 1 and 10 mm (ColN^2-19^ and ColE9^2-18^, respectively) although, the concentration of peptides is not exact due to the lack of Tyr and Trp. Binding was analyzed using the manufacturer's software.

### Single channel conductance measurements

Electrical measurements were performed in 100 mm potassium chloride, 20 mm potassium phosphate, pH 6.5, at room temperature. A 25-μm thick Teflon film (Goodfellow) with an aperture of ∼100 μm large diameter was placed between the two 1-ml Delrin compartments separating *cis* (grounded) and *trans* chambers (36). The chambers were connected using Ag/AgCl electrodes into a patch clamp amplifier (Axopatch 200B) linked to a head stage (CV203BU), both from Axon Instruments. The aperture was initially coated with 1% (v/v) hexadecane in pentane and after the chambers were filled with buffer, a lipid bilayer was formed using 2 µl of 5 mg/ml of 1,2-diphytanoyl-*sn*-glycerol-3-phosphocholine (Avanti Polar Lipids) dissolved in 10% (v/v) pentane. The insertion of a single OmpF trimer into the lipid bilayer was performed by the addition of 8 nm final OmpF concentration into the *cis* compartment. A potential of ±200-300 mV was applied to aid in protein insertion. The determination of positive or negative OmpF channel asymmetry was performed, using the I/V curve, as previously described in Ref. 31. The data were collected upon the addition of 1 μm ColN^2-19^ peptide into either *cis* or *trans* sides, whereas voltage of −100 or +100 was applied from the *trans* side. The amplified signal was filtered at 2 kHz and sampled at 10 kHz using Digidata 1440A digitizer (Axon Instruments). The data were analyzed as previously reported (13) using pClamp software (Molecular Devices).

### Widefield microscopy

The following *E. coli* strains were used for microscopy: BW25113 (WT), JW0912-1 (Δ*ompF*), JW2203-1 (Δ*ompC*), and BZB1107 (Δ*ompF*, Δ*ompC,* Δ*lamB*). A day prior to microscopy single cultures of each strain were used to inoculate 10 ml of LB and grown for 8 h at 37 °C. 500-µl pellets of each culture were transferred into M9-glucose (2 mm MgSO_4_, 0.1 mm CaCl_2_, 0.4% (w/v) d-glucose) overnight. After overnight growth 500-µl pellets of each culture were transferred into 4-ml aliquots of fresh M9-glucose supplemented with 30 µg/ml of kanamycin, where appropriate. Cells were grown to an OD_600_ of 0.25–0.6. For each culture an equivalent of 500 µl of OD_600_ 0.6 cells were pelleted and labeled with 200 nm ColN^1-185^-mCherry for 10 min. After labeling, cells were fixed in 4 °C, 4% formaldehyde (diluent: PBS) for 30 min. After fixing strains, JW0912-1 and BZB1107 were washed once in PBS and all other strains were washed twice. After final wash steps all strains were resuspended in 40 µl of PBS and 7.2-µl aliquots of resuspended cells were placed onto 1% agarose pads (diluent: PBS). All imaging was conducted on the ONI: Nanoimager S. A 561-nm laser line was used at 20% power to visualize ColN^1-185^-mCherry labeled OmpF. For each field of view 100 frames were collected at an exposure of 100 ms.

### Widefield microscopy analysis

For each field of view the 100 frames collected were averaged, cells were segmented using a transillumination image, for fluorescence intensity experiments the *n* number therefore represents both individual cells and clumps of multiple cells. Custom written python scripts, available upon request, were used to calculate the average cell background subtracted fluorescence intensity.

### Fluorescence recovery after photobleaching

For FRAP experiments, New England Biolabs BL21(DE3) cells were prepared as described for widefield microscopy experiments. Imaging was conducted on a Zeiss LSM780. Cells were imaged with a 2% 561-nm laser line with a 100 ×1.4 NA lens, bleaching was conducted by exposing a 70 × 30 px region to 100% power for 15 iterations. Images were collected every 4 s for a total of 120 s, a pinhole diameter of 90 μm was used, channel gain was set between 800 and 900, light was collected between wavelengths of 578 and 696 nm. FRAP curves were generated by measuring average fluorescence intensities in three 50 × 50 px regions in each stack: target area (tA), reference area (rA,) and background area (bA). The relative fluorescence recovery was calculated for the stack using the following equation.

(Eq. 1) $$\left(tA_{n}-bA_{n}\right)\times \left(\frac{rA_{0}-bA_{0}}{rA_{n}-bA_{n}}\right)$$

Where n is the frame to be analyzed and 0 is the pre-bleach frame. The FRAP curve was then normalized so that the pre-bleach relative fluorescence intensity was 100%.

### Native MS

A complex of 10 μm OmpF and 10 μm ColN was prepared in 3 ml of 100 mm ammonium acetate, pH 6.9, 1% β-OG. 20 µg of sequencing grade trypsin (Promega) was added and incubated at 37 °C for 2 h. Digested complex was concentrated to 500 µl using a VivaSpin4 with 30-kDa membrane before purification on a 10/300 S200 analytical gel filtration column equilibrated in 100 mm ammonium acetate, 1% β-OG. Fractions containing OmpF were analyzed by native MS. Native MS Protein aliquots (50 µl of 20 μm) were desalted prior to native MS analysis using biospin-6 (Bio-Rad) columns equilibrated in 200 mm ammonium acetate supplemented with 1% β-OG detergent. Samples were loaded (3 μl) into gold-coated silica capillaries prepared in-house using a procedure described previously. Capillaries were mounted to a static spray block of a quadrupole–TOF (qTOF) mass spectrometer (micromass, UK), modified for high-mass transmission, and spray was induced by applying 1,700 V to the capillary. Pressure was maintained at ∼7.3 × 10^−3^ mbar in the source region of the instrument, necessary to improve transmission of protein complexes. An acceleration voltage of 200 V was applied to the sample cone and 200 V to the collision cell to remove β-OG micelles and liberate the OmpF-ColN peptide complex. Data were processed using Masslynx software. Spectra were smoothed (×2) using the mean smoothing method with a smooth window of 10 and baseline subtracted using a polynomial order of 1 (20% below curve).

### Killing assays

The B^E^3000 *E. coli* cell line was streaked on a LB-agar plate and grown overnight at 37 °C. The next day, 5 ml of LB was inoculated from a single colony and grown until OD_600_ reached 0.6. The bacterial cells were then used to inoculate LB with 0.7% (w/v) agar that was melted and equilibrated to 45 °C. After the plate containing soft agar was cooled and set, it was spotted with 3-fold serial dilutions of purified proteins ranging from 10 μm to 57 pm. The inverted plate was grown overnight at 37 °C and was evaluated for zones of clearance in the bacterial lawn the next morning.

### Immunodiffusion assay

The immunodiffusion assay was performed as previously reported (37) using 0.5% agarose gel that was melted in potassium phosphate buffer, pH 6.65, containing 1% (w/v) β-OG. Approximately 4 ml of gel was poured into a 35-mm Petri dish. After the gel solidified, 4 wells with 3-mm diameters were carved out. 20 µl of protein at 17 μm was added to each well. The plate was incubated overnight at room temperature to allow diffusion of proteins. The precipitated bands between two protein species were observed the next morning.

### SDS-PAGE and LPS staining

12% Polyacrylamide SDS-PAGE was run for ∼30 min at a constant current of 30 mA after the samples were boiled in loading dye for 5 min, LPS staining was performed using Pro-Q Emerald 300 LPS gel stain kit P20495 from Life Technologies Ltd. Rough LPS from *E.coli* J5-Rc mutant (Sigma-Aldrich) was used as a standard.

## Data availability

All data presented are available on request from the corresponding author.

## Supplementary Material

Supporting Information
