# Supplementary material for: Bifurcated binding of the OmpF receptor underpins import of the bacteriocin colicin N into Escherichia coli
Source: J Biol Chem. 2020 May 12;295(27):9147–56. doi: 10.1074/jbc.RA120.013508 (PMC7335789; doi:10.1074/jbc.RA120.013508)
Supplement: Supporting Information [file supp_295_27_9147__index.html]

Bifurcated binding of the OmpF receptor underpins import of the bacteriocin colicin N into Escherichia coli — Bifurcated OmpF binding by Colicin N — Bifurcated binding of the OmpF receptor underpins import of the bacteriocin colicin N into Escherichia coli — Bifurcated OmpF binding by colicin N — Supporting Information 

# Bifurcated binding of the OmpF receptor underpins import of the bacteriocin colicin N into *Escherichia coli*

## Supporting Information

- Supporting Information (to be published online) - Supplementary figures 1 and 2 and their associated figure legends
