# Supplementary material for: Bifurcated binding of the OmpF receptor underpins import of the bacteriocin colicin N into Escherichia coli
Source: J Biol Chem. 2020 May 12;295(27):9147–56. doi: 10.1074/jbc.RA120.013508 (PMC7335789; doi:10.1074/jbc.RA120.013508)
Supplement: Supporting Information [file supp_RA120.013508_159578_2_supp_525485_q9x0j0.pdf]

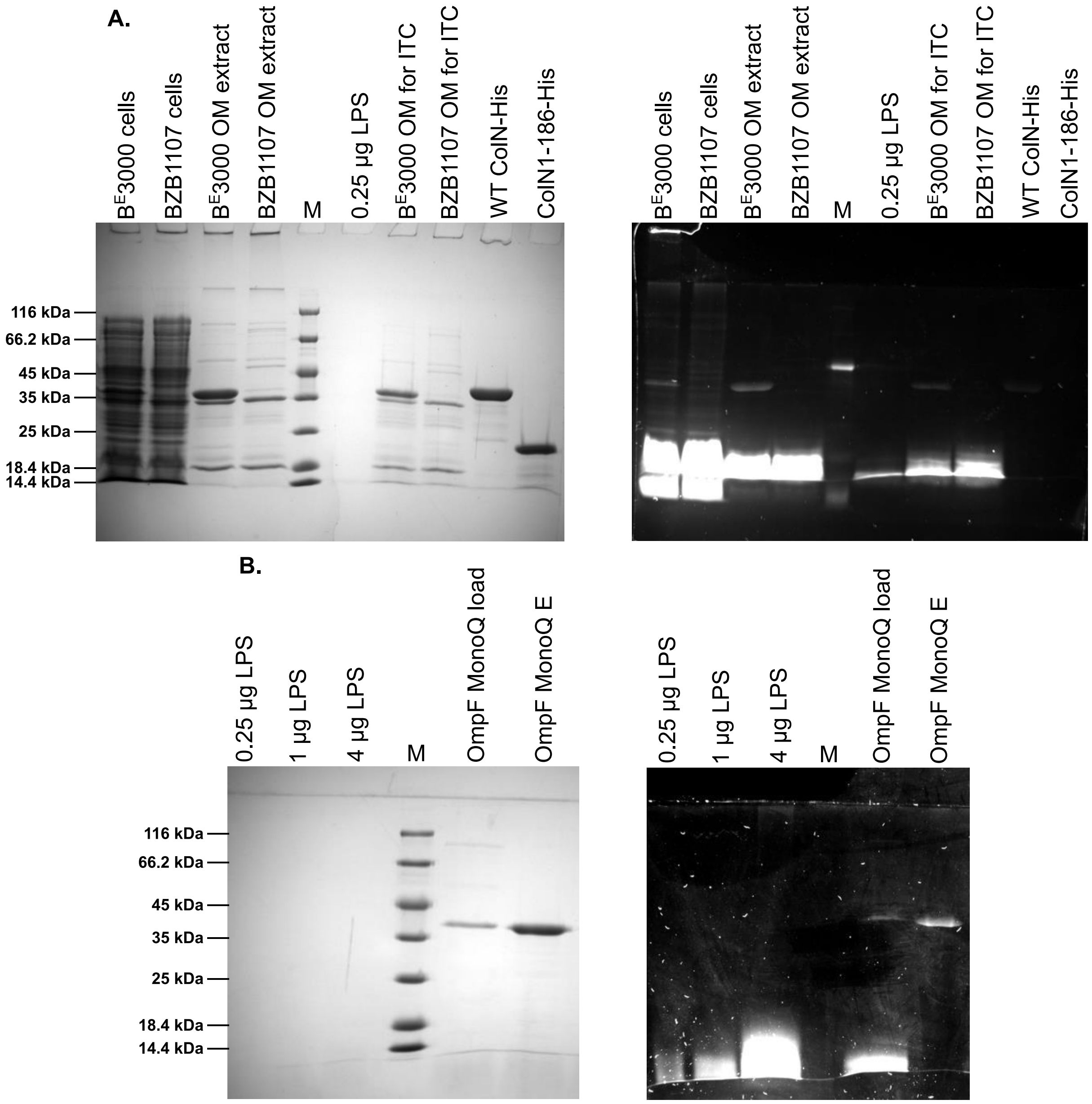

Supplementary Figure 1. (A) SDS-PAGE analysis of whole cell lysate, and purified membranes from *E. coli* strains used for ITC experiments in this study. Left: coomassie blue staining, Right: LPS staining. (B) SDS-PAGE analysis of purified OmpF used in this study. Left: coomassie blue staining, Right: LPS staining.

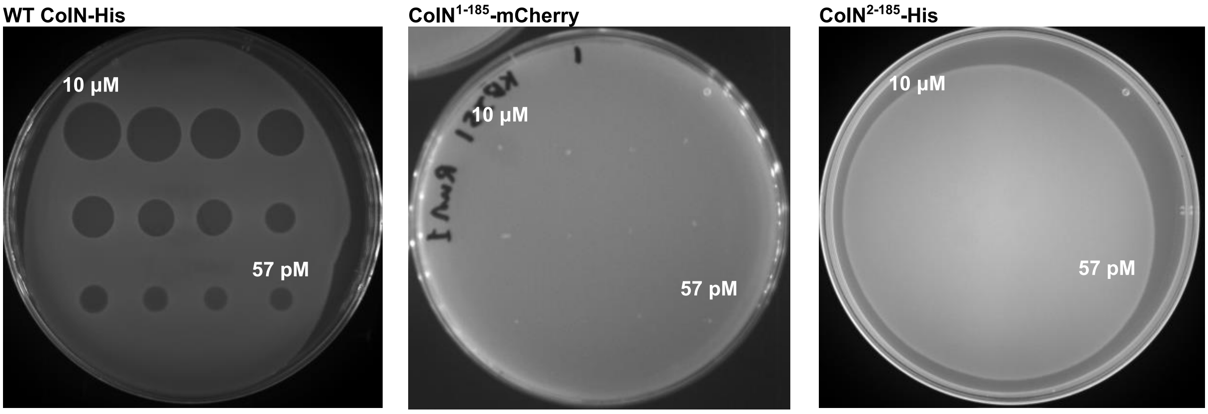

Supplementary Figure 2. Colicin N killing assays for three constructs. 3 fold serial dilutions, starting at 10 μM, down to 57 pM of each construct were spotted onto LB-Agar plates containing *E. coli* B^{E}3000 . WT Colicin N with a C-terminal His-tag displays killing down to 57 pM concentrations. ColN$^{1-185}$-mCherry and ColN$^{2-185}$-His both show a lack of any killing activity.
